# SOCIAL SUPPORT FROM FRIENDS AND INCIDENCE OF COGNITIVE IMPAIRMENT A DECADE LATER

**DOI:** 10.1093/geroni/igac059.464

**Published:** 2022-12-20

**Authors:** Sara Moorman, Manacy Pai

**Affiliations:** Boston College, Chestnut Hill, Massachusetts, United States; Kent State University, Kent, Ohio, United States

## Abstract

We inquire how diverse forms of social support from non-kin are associated with cognitive aging across ten years. We analyze data from 4,687 participants in the 2011 and 2021 waves of the Wisconsin Longitudinal Study (WLS), a cohort study of Wisconsin adolescents from the birth cohort of 1939 and their siblings. Net of sex, educational attainment, income, smoker status, depressive symptoms, self-reported health, diabetes, heart disease, high blood pressure, high cholesterol, and stroke incidence, each additional domain in which participants perceived that they had instrumental support available from friends in 2011 was associated with a 0.07 standard deviation increase in cognitive function (i.e., TICS-m score) a decade later (p = .001). Moreover, participants who had a non-kin confidante in 2011 had significantly lower odds of mild cognitive impairment (MCI) in 2021 (OR = 0.57; p = .006). Robust social engagement may help protect and maintain older adults’ cognitive skills.

